# Development and evaluation of an intervention aiming to reduce fatigue in airline pilots: design of a randomised controlled trial

**DOI:** 10.1186/1471-2458-13-776

**Published:** 2013-08-26

**Authors:** Alwin van Drongelen, Allard J van der Beek, Hynek Hlobil, Tjabe Smid, Cécile RL Boot

**Affiliations:** 1 Department of Public and Occupational Health, EMGO Institute for Health and Care Research, VU University Medical Center, PO Box 7057, Amsterdam 1007, MB, the Netherlands; 2 KLM Health Services, Schiphol Airport, the Netherlands; 3 Body@Work TNO VUmc, Research Center on Physical Activity, Work and Health, VU University Medical Center, Amsterdam, the Netherlands

**Keywords:** Flight crew, Pilots, Irregular working hours, Fatigue, Intervention, Tailored advice, Implementation, Smartphone application, mHealth

## Abstract

**Background:**

A considerable percentage of flight crew reports to be fatigued regularly. This is partly caused by irregular and long working hours and the crossing of time zones. It has been shown that persistent fatigue can lead to health problems, impaired performance during work, and a decreased work-private life balance. It is hypothesized that an intervention consisting of tailored advice regarding exposure to daylight, optimising sleep, physical activity, and nutrition will lead to a reduction of fatigue in airline pilots compared to a control group, which receives a minimal intervention with standard available information.

**Methods/design:**

The study population will consist of pilots of a large airline company. All pilots who posses a smartphone or tablet, and who are not on sick leave for more than four weeks at the moment of recruitment, will be eligible for participation.

In a two-armed randomised controlled trial, participants will be allocated to an intervention group that will receive the tailored advice to optimise exposure to daylight, sleep, physical activity and nutrition, and a control group that will receive standard available information. The intervention will be applied using a smartphone application and a website, and will be tailored on flight- and participant-specific characteristics. The primary outcome of the study is perceived fatigue. Secondary outcomes are need for recovery, duration and quality of sleep, dietary and physical activity behaviours, work-private life balance, general health, and sickness absence. A process evaluation will be conducted as well. Outcomes will be measured at baseline and at three and six months after baseline.

**Discussion:**

This paper describes the development of an intervention for airline pilots, consisting of tailored advice (on exposure to daylight and sleep-, physical activity, and nutrition) applied into a smartphone application. Further, the paper describes the design of the randomised controlled trial evaluating the effect of the intervention on fatigue, health and sickness absence. If proven effective, the intervention can be applied as a new and practical tool in fatigue management. Results are expected at the end of 2013.

**Trial registration:**

Netherlands Trial Register: NTR2722

## Background

Long and irregular working hours as well as crossing multiple time zones are common working conditions of flight crew [1]. These conditions can cause travel fatigue, reduce sleep quality and quantity, and disrupt the circadian rhythm. The latter can lead to jet lag symptoms as well [1,2]. All factors may contribute to increased fatigue. Prolonged fatigue has been shown to cause health problems, impaired performance capability and a disturbed work-private life balance [3]. Furthermore, it has become clear that long term exposure to reduced sleep and circadian disruption can lead to cardiovascular diseases, gastrointestinal disorders, and cancer [4-6]. More recently, this exposure has also been related to body weight gain, metabolic syndrome, and diabetes [7-9].

Among flight crew, fatigue is experienced regularly [10]. In a study conducted in New Zealand for instance, 64% of the participating pilots reported to be fatigued at least once a week due to their working hours [11]. In comparison, in a Dutch cohort study of a general working population, the prevalence of fatigue due to work was found to be 22%. Another study among pilots showed that 75% of them acknowledged fatigue as a serious problem during their job. Further, 71% of them admit to have been dozed off at least once during a flight [1].

In recent years, more knowledge has become available about the influencing factors on disturbance of the circadian rhythm, and possible countermeasures of fatigue [12-14]. It has been shown that by correct timing of exposure to and avoidance of daylight, the most important biorhythm synchronizer (or zeitgeber) [2,12,13], jet lag symptoms can be reduced. Additionally, an optimal timing and duration of sleep can reduce the disturbance of the biological clock and thereby, reduce fatigue [2,13,14]. Further, correct timing of certain types of physical activity can enhance sleep duration and quality [3]. Moreover, the intake or avoidance of food may diminish jet lag symptoms during certain phases of the sleep/wake cycle, and caffeine can temporarily alleviate fatigue [3,12-14]. Additionally, the specific macronutrient composition of meals has shown to be able to stimulate either alertness or relaxation [15,16]. Based on these findings, several measures have been proposed to counter the negative effects of the aforementioned working conditions of flight crew.

These general measures should be translated into practical advice to enhance implementation. For flight crew, the specific advice depends on several variables (e.g. flight direction, flight duration, and number of time zones crossed). This implies that the advice differs per destination and person (e.g. morning vs. evening types), and that the total number of different advices is high. Due to this complexity, translating the theoretical knowledge into training programs for flight crew has proven to be difficult [2,17]. Airlines and authorities sometimes provide employees with fatigue-related information at the start of their job, but most often continued education is not assured. And although aircraft companies such as Boeing and Airbus developed training modules aiming to reduce fatigue, the effects of these programs have hardly been examined systematically [17,18]. The few studies that did study the effects of such training programs, reported some effects [19,20] but they combined their training programs with alterations in work schedules, so that it was impossible to address the measured fatigue reduction and performance improvement to the training program alone. Other studies did find improved knowledge, awareness [21], layover sleep, and in-flight alertness [22] after short term application of fatigue management advice in flight crew, but did not measure the long term effects.

Further, in the aforementioned studies, advice was mostly transferred through the use of paper or in the form of books or instruction materials. This kind of implementation has led to low compliance, which was shown in an article by Flower [23]. Their advice cards program among British Airways pilots was received positively, but the compliance of 40% proved to be low. Therefore, it might be possible that the lack of compliance and outcomes in transferring fatigue-related knowledge to flight crew is (partly) due to the chosen medium. There might be a solution for this implementation problem because in more recent health behaviour literature, the large potential of the use of new media sources, such as computers and internet, has become clear [24,25]. It has been stated that the use of web-based interventions is more effective in increasing health behaviour knowledge compared to non-web-based interventions [26]. Further, to improve adherence, these interventions should be designed to allow individuals to tailor it to their own specific needs. This so called tailoring can significantly affect behaviour regarding safety, smoking, physical activity and dietary intake [24,27,28].

In summary, it can be stated that it is still largely unclear what the effects are of tailored advice regarding exposure to daylight, sleep, physical activity, and nutrition on fatigue of flight crew. Therefore, this paper describes the development of an intervention consisting of tailored advice for airline pilots, and the design of the randomised controlled trial evaluating the effect of the intervention on fatigue, health and sickness absence. The advices are evidence-based and aim to optimise the pilots’ behaviour with regard to:

•Exposure to daylight (timing and duration)

•Sleep (sleep behaviour and timing of sleep)

•Nutrition (dietary behaviour and timing of dietary behaviour)

•Physical activity (form and timing of physical activity)

It is hypothesized that an intervention consisting of easy obtainable tailored advice, will lead to a significant reduction of fatigue in airline pilots compared to a minimal intervention, based on standard fatigue related information available within the airline company.

## Methods/design

An intervention has been developed and will be evaluated by means of a two-armed randomised controlled trial (RCT). The intervention, named MORE Energy, will have a follow-up period of six months. The recruitment of the participants will start in autumn 2012. The study design and procedures have been assessed by the Medical Ethics Committee of the VU University Medical Center, Amsterdam, the Netherlands (#2011/065).

### Study population

The study population will consist of pilots of all aircraft types of a large internationally operating airline company.

### Study procedures

**Figure 1:**
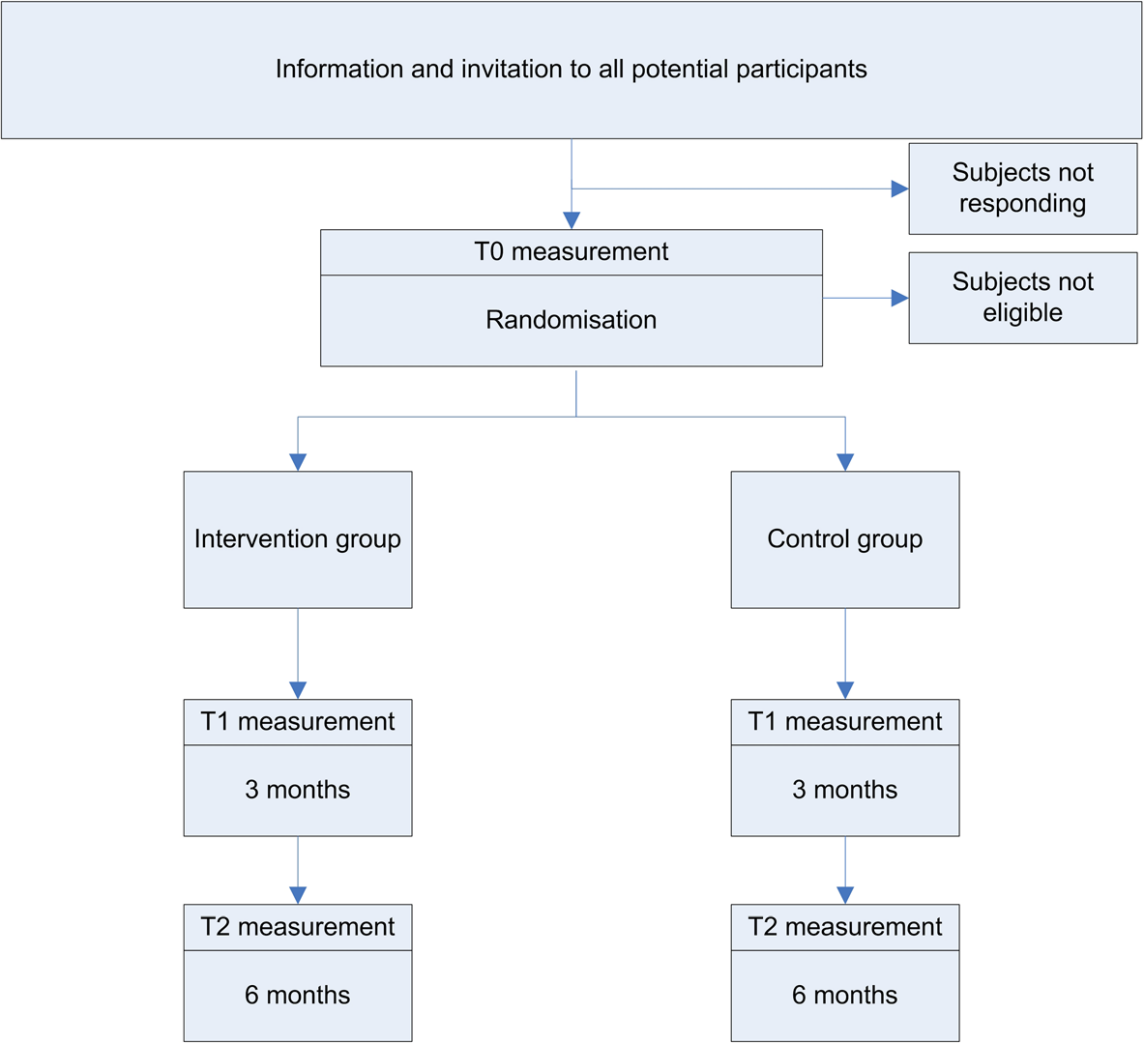
Study design.

The study design is presented in Figure 1. First, potential participants will be made aware of the project by means of a publicity campaign, using intranet, internet and news bulletins. In addition, meetings with supervisors will be held to make sure they can propagate the study to their employees, and motivate them to participate.

After the publicity campaign, all pilots will receive a postal information brochure. In this brochure, the pilots will be made aware of the participation mail, which they will receive in their company e-mail inbox. In this e-mail, all potential participants will be asked to fill in the baseline questionnaire, by using the included unique internet link. By filling in the questionnaire the pilots agree to participate in the study, no additional written informed consent will be obtained. After the researchers receive the filled in questionnaire, participants will be allocated to either the control or intervention group.

### Inclusion and exclusion criteria

The pilots can participate in the study if they:

•are not on sick leave for more than four weeks at the moment of recruitment;

•own a smartphone or tablet with an Android or iOS (iPhone/iPad) operating system.

### Randomisation

Study group allocation will be conducted at individual level, using the minimisation technique. After the baseline measurement the participants will be assigned to one of the two study groups. A minimisation procedure will make sure that the group allocation of the next participant enrolled in the trial takes into account the characteristics of those participants already enrolled. The aim is that each allocation should minimise the imbalance across multiple factors [29]. The factors that are considered for minimisation are aircraft type (five different types of aircraft units), and job title (captain, first officer, and second officer).

### Development of the intervention

**Figure 2:**
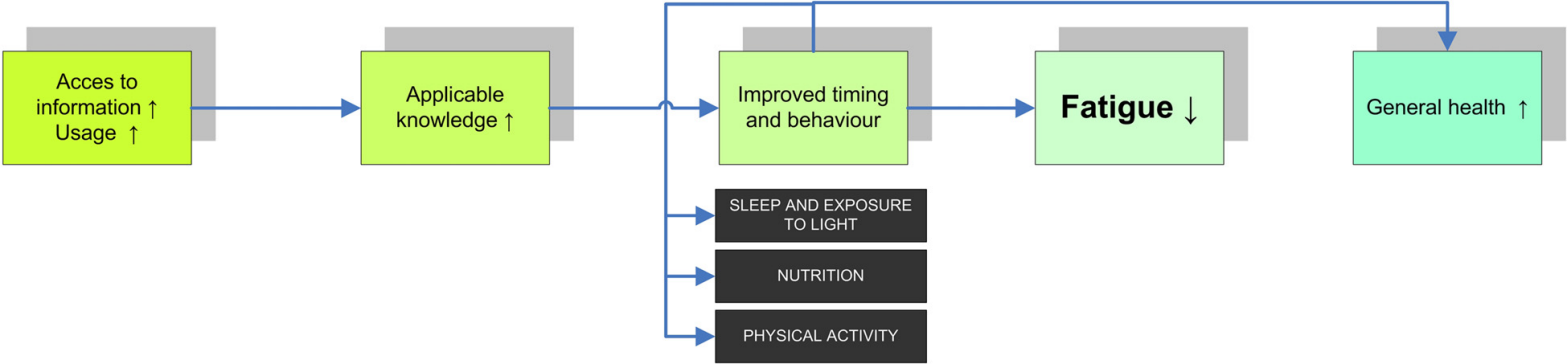
Intervention objectives.

To optimize the content of the advices, a literature study was performed in order to gain insight in the latest scientific knowledge regarding work-related fatigue in flight crew. Based on this literature study, the following intervention objectives were defined (Figure 2).

It is hypothesized that tailoring the relevant fatigue-related information, and making it easy accessible to the flight crew, will lead to improvement of their knowledge about the different aspects. This will be measured using knowledge tests before and during the intervention. Improved knowledge will enlarge the possibility that the participant will use this knowledge to improve their behaviour regarding exposure to daylight, sleep, physical activity and nutrition, before, during, and after flight schedules. Accordingly, it is hypothesized that if their behaviour is altered, participants’ circadian disruption and fatigue will reduce, leading to an improvement of general health.

To find out what implementation strategy and what medium should be used to optimise compliance to the intervention, focus groups with the target population were held. For each aircraft type, two focus groups were organised. Considering five different types of aircrafts, ten focus groups were held. A total of 30 pilots attended the focus groups, heterogeneous regarding age, gender and job title. The discussions were recorded and field notes were written. The focus groups made clear that the intervention should be easy available, appealing, and to be used by pilots of all ages and ranks. Further, it was made clear that the intervention should be evaluated by means of a test phase first, and that it should be evident that the advice given is evidence based. The advices should be made flight schedule specific; short haul pilots have a different need for information and advice than long haul pilots have.

The input from these meetings was used to elaborate the intervention strategy and to take into account the raised facilitators and barriers for implementation. Finally, interviews with key management stakeholders were held, aiming to match the intervention to the present laws and legislation, collective labour agreement, and the policy of the airline company.

After the development of the intervention, it was pre-tested by pilots and researchers. The group of test pilots was heterogeneous with respect to both aircraft units and job type. In total, 34 pilots were invited to take part in this test phase, which lasted eight weeks in total. At the end of this period, the pilots who had actually participated were asked to fill in a digital evaluation form. Further, they were asked if they were available for a telephone consultation, in case the researchers needed additional information from them. Based on the results of the test phase evaluation, the intervention was reconsidered and optimised where necessary. An important result of the test phase was the report of the short-haul pilots that their advice was not substantive enough. This issue was addressed by adding more extensive advices with regard to short-haul flight schedules.

### Description of the intervention

As a result of the focus groups, a tailoring strategy was constructed. It was decided that the best tool to transfer the knowledge would be using a smartphone application. This ensures that the advice can be supplied tailored to flight characteristics (e.g. flight direction, departure and return time, number of time zones crossed) and to personal characteristics (morning vs. evening types).

For each destination with its specific schedule, the participants are provided with specific advices on optimal sleep, nutrition, and physical activity, in order to reduce fatigue. The user can fill in his flight and location, after which he will receive the advices. Subsequently, the user can choose himself to read extensive information about either exposure to daylight, sleep, nutrition, or physical activity in the glossary menu. For each screen of the application, a help menu is available.

A website containing more background information was developed alongside the smartphone app. In the app, the user is guided to the website to read, listen to and see more detailed information. See Additional file 1 for examples of the application and website.

Participants in the intervention group will be stimulated to consider the advice on the application and the website by means of reminders. The first form of reminding occurs through timed alerts; the user receives an alert once he has not opened the app for longer than three weeks. Another reminder strategy that will be used is called geofencing; the user is given an alert each time he is outside the Netherlands, with a maximum of one alert per four days.

#### Control intervention

The participants allocated to the control group will receive a minimal intervention consisting of the available fatigue-related information of the airline company. This information is normally scattered throughout the company and by putting it to the study website it is made sure that the information is available for each participant. Minimal intervention strategies are commonly used for control groups in order to compare them to an intervention group [30].

Consequently, the researchers will send an e-mail to the participants with instructions how to obtain and use the advices. The members of the intervention group will receive a link to download the smartphone application and a link to access the intervention website. The participants of the control group will be invited to log in to the study website, after which they will be directed to the standard available information. During the intervention period, all participants will be kept involved by sending a regular newsletter with information about the study in general, and upcoming questionnaires. Reminders will be sent to increase compliance among all participants, and communication throughout the airline company will be used to raise awareness.

### Outcomes

Measurements will take place at three moments; participants will be asked to fill in questionnaires at baseline (T0), and at three (T1), and six months after baseline (T2). Before each follow-up measurement, subjects from both groups will receive an e-mail in which they are asked to fill out the online questionnaires. Reminders will be sent to increase the response rate. All questionnaires are in Dutch.

#### Primary outcome

The primary outcome variable is fatigue. Fatigue will be measured using the 20-item Checklist Individual Strength (CIS) [31]. The questionnaire consists of four dimensions; the subjective experience of fatigue, reduction in motivation, reduction in activity, and reduction in concentration. It has been found that the questionnaire has a good internal consistency: the Cronbach’s α for the total CIS was 0.90 and for the scales the α ranged from 0.83 to 0.92 [31].

The questions have seven answer options, ranging from “yes that is correct” to “no that is not correct”.

#### Secondary outcomes

•*Sleep*

Sleep quantity and quality as well as timing of sleep will be measured using the Jenkins Sleep Scale [32] and the subscales subjective sleep quality, sleep latency, sleep duration, and use of sleeping medication of the Pittsburgh Sleep Quality Index [33].

The Jenkins sleep scale is a short, 4-item, multiple choice questionnaire, which is regularly used in flight personnel [34]. The internal consistency has been found to be reasonable, with a Cronbach’s α of 0.79. The used subscales from the Pittsburgh Sleep Quality Index comprise 5 multiple choice items. The total component scores of the PSQI proved to have an overall Cronbach’s α of 0.83, indicating a good internal consistency.

•*Nutrition*

Nutritional behaviour will be measured using a self-developed questionnaire. The questionnaire contains 13 questions concerning the regularity of meals taken, snacking, usage of breakfast, composition of meals, and drinking habits. The questionnaire comprises different types of questions, both open and multiple choice.

•*Physical activity*

The amount of physical activity during leisure time will be measured using questions on the recommended quantity for physical activity and exercise in healthy adults [35,36]. These measurements are often used in RCTs and comprise two open-ended questions.

•*Need for recovery*

Need for recovery will be measured using the 11-item ‘need for recovery scale’ from the Dutch Questionnaire on the Experience and Evaluation of Work (Dutch abbreviation VBBA), which has shown to be valid and reliable, and to have a good internal consistency of 0.88 [37]. All questions have two answering categories (yes / no).

•*Sickness absence*

Data on sickness absence (number of absence days and number of spells), will be collected from the records of the Occupational Health Service and Human Resource department of the airline company.

•*Work-private life balance*

Work-private life balance will be measured using the shortened version of the SWING questionnaire [38]. This 17-item questionnaire has been developed to measure and distinguish four types of interaction; negative work-home interference, positive work-home interference, negative home-work interference, and positive home-work interference. It has been shown that the questionnaire measures all constructs reliably, with Cronbach’s α’s ranging from 0.75 to 0.84 [39].

•*General Health*

General perceived health will be measured using two questions of the Dutch version of the SF-36 Health Survey which have proven to be moderately valid and reliable (0.81) [40].

•*Knowledge*

During each measurement, the knowledge of the participants regarding the relevant fatigue-related information will be measured by means of 20 true or false statements, containing all sub-domains (exposure to daylight, sleeping, physical activity, and nutrition) of the intervention.

•*Body Mass Index (BMI)*

Participants will be asked for their body height and body weight. Afterwards BMI will be computed as body weight divided by the square of height (kg/m^2^).

#### Other study parameters

•*Socio-demographic variables*

At baseline, socio-demographic data (age, gender, job title, flight unit, and household composition) will be collected.

•*Morningness-eveningness.*

At baseline, personal morningness-eveningness preference will be measured using the VOA, the Dutch version of the Morningness-Eveningness Scale (MEQ) [41].

•*Tobacco and alcohol*

Smoking tobacco and alcohol consumption are potential effect modifiers. Therefore, tobacco smoking behaviour and alcohol consumption will be assessed at baseline.

•*Chronic disease*

Having any kind of chronic disease might also be acting as an effect modifier. Therefore having a chronic disease will be measured by one yes or no question: “do you have a chronic disease?”

### Process evaluation

Besides the effect evaluation, the process of the implementation of the intervention will be evaluated, in accordance with the Steckler&Linnan framework [42]. During the intervention period, an extensive userlog will be kept, and the delivery of the intervention will be quantified. Besides, the extent to which participants use the intervention will be objectively measured through the control management system (CMS) of the application. This system will measure the number of times the intervention participants will request an advice, and, each month, all data about usage will be stored in a database. Likewise, the number and duration of all the participants’ visits to the study website will be stored as well.

Using the questionnaires, the participants will be asked if they have read and used the advices, and if they think their behaviour (regarding exposure to daylight, sleep, physical activity and nutrition) and their timing of this behaviour, has changed due to the advices. Further, they will be asked about their satisfaction with the intervention program, concerning both the smartphone application and the website.

### Statistical analysis

To investigate the success of the randomisation procedure, potential confounders or effect modifiers (age, gender, type of aircraft flown, household composition, morningness-eveningness) will be compared between the intervention and control group by Student t-tests for independent samples and Chi square tests (χ2).

The effectiveness of the intervention (time*group interaction) will be analysed using linear mixed model analyses with the outcome measures at follow-up (T1-T2) as the dependent variables. The dependent variables are fatigue, sleep, physical activity, nutrition, need for recovery, sickness absence, work-private life balance, general health and knowledge, whereas research condition (intervention or control group) is the independent variable. Possible confounding or mediating factors will be considered.

A detailed analysis plan will be developed prior to finalisation of the dataset. For all analyses a two-tailed significance level of <0.05 will be considered statistically significant. The analyses will be performed using PASW 20.0 (SPSS Inc. Chicago, Illinois, USA).

### Sample size

The sample size is based on finding an effect on the primary outcome of the intervention, perceived fatigue. Fatigue will be measured using the 20-item CIS questionnaire [43]. The mean total score on this questionnaire for healthy employees is 47.3 (SD = 19.8) according to Beurskens et al. [43] (score range 0–140). Power calculations indicate that to detect a relevant 10% difference in fatigue, 246 subjects are necessary in each study group (with power = 0.80 and alpha = 0.05). Taking the expected loss to follow-up (25%) into account, a sample size of approximately 656 employees is required.

## Discussion

This paper describes the development of an intervention consisting of a set of tailored advices (about daylight exposure, sleep, physical activity, and nutrition) delivered through a smartphone application for airline pilots, and the design of a randomised controlled trial evaluating its effect on fatigue, health and sickness absence.

Many authors have called for additional efforts to prevent the detrimental health effects of irregular working hours among the employees involved [9,44]. Fortunately, knowledge about the influencing factors of fatigue and disturbance of the circadian rhythm is available. Since educational programs in the past generally did not show a long term fatigue reduction among flight crew, the present study tailors the advices and makes them easy obtainable. By using new forms of media, it aims to improve health behaviour, to reduce experienced fatigue, and to improve general health.

### Strengths & limitations

The fact that this intervention uses a smartphone application to transfer knowledge and aims to change behaviour is a strength. A recent estimation in the Netherlands showed that 52% of all phone users own a smartphone and this number is expected to grow to 65% in 2012 [45]. Moreover, in the specific target population of this study the percentage of smartphone users is expected to be even higher. Smartphones and tablet computers have several advantages; they are easy to use and to carry on, and do not necessarily need to have access to internet in order to work as an information source. Further, the technology of the smartphone application itself makes it possible that the advices are presented specific per function, morningness-eveningness type, and flight schedule. Evaluating smartphone apps to improve health behaviour by using a RCT is relatively new. To our knowledge, the study of Quinn et al. [46] was the first to present results of a RCT on mobile health (mHealth). In their study, positive effects were found on the control of blood glucose in diabetes patients. The number of mHealth studies is expected to rise in the upcoming years since several research protocols have been published [47,48]. Also a guideline for both eHealth (web-based interventions to improve health behaviour) and mHealth RCTs was published recently [49].

The MORE Energy study is the first randomised controlled trial that uses mHealth to improve health behaviour in flight personnel. Advantages of mHealth are partly similar to those of eHealth, and include the unique characteristics of technology (e.g. video transmission, interactiveness), its relative cost-efficiency, the immediate and easy access of information or advice, and the flexibility of user control of the intervention [50]. However, the evaluations of these kind of interventions are often complicated by the fact that a substantial proportion of participants may have dropped out because of non-use or loss to follow-up [26,49]. Because the effectiveness is dependent on participants actually using the intervention, in the current study, the usage of the application and website will be measured for each participant. Furthermore, the process evaluation will give insight in compliance, how the intervention was evaluated by participants, and how the advice was used. By tailoring the intervention to the needs of the target population through focus group discussions, it is expected that the risk for low compliance in this intervention is somewhat reduced.

The use of focus groups with the target population is also a strength. Together with the interviews with the key management stakeholders of the company, it resulted in an implementation strategy that took into account a lot of barriers faced by the target population. With this strategy, it is thought that problems faced by similar studies [19] are partly overcome. These studies used other kinds of media to transfer knowledge, and were therefore limited in their capability to tailor their advice. In the MORE Energy study, advice will not only be tailored, but will be offered in such a way that participants can choose the advices that fulfil their specific needs at specific moments.

There are possible limitations to this study as well. Firstly, results from earlier studies and indications from the focus groups point out that there might be a substantial group of pilots that will not participate because they have their own long-lasting routine to cope with flight schedules, and are not open for new insights. This will lead to selection bias [51]. Further, because the intervention will use a smartphone application, employees who do not own such a device are excluded from this study. Therefore, even though smartphones and tablets are widely used throughout pilots, a certain subgroup of employees will not be able to participate. Unfortunately, since we do not have information about who does or does not own a smartphone, we can only compare the demographic characteristics of the whole population of airline pilots with those of the participants.

Another limitation of the study is the possible crossover of information. It was decided to randomize the participants at an individual level. This means that, during a flight routine a control group pilot and an intervention group pilot with smartphone application may share the cockpit which makes contamination plausible. Because the application is personalised and both the application and website have a unique login per participant, it is assumed that contamination will have a limited effect on the resulting differences in outcome between groups though. Finally, a possible limitation is that the participants will not be blinded for design. Because the control group will receive a minimal intervention only, and the intervention group will receive extensive, specific and tailored advice (which is easily accessible through both smartphone application and website) the contrast between groups is considered sufficient however. Although we chose to offer the control group a minimal intervention rather than no intervention, more attention was given to the intervention group. Because of this, the Hawthorne effect cannot be fully excluded.

This RCT will investigate whether a tailored mobile health intervention can reduce fatigue and improve general health of airline pilots by improving the knowledge and behaviour with respect to exposure to daylight, sleep, nutrition and physical activity. If results are positive, it would provide a new tool in fatigue management, possibly reducing the health risks of irregular working times of both flight crew and shift workers. Results of the MORE Energy study will become available at the end of 2013.

## Competing interests

The authors declare that they have no competing interests.

## Authors’ contributions

All authors contributed to the design of the study and the intervention. AD wrote the manuscript, which was commented on by all authors. All authors have read and approved the final version of the manuscript.

## Pre-publication history

The pre-publication history for this paper can be accessed here:

http://www.biomedcentral.com/1471-2458/13/776/prepub

## Supplementary Material

Additional file 1 Examples of the MORE Energy application and website. Click here for file
