# Supplementary figures and images for: Development and evaluation of an intervention aiming to reduce fatigue in airline pilots: design of a randomised controlled trial
Source: BMC Public Health. 2013 Aug 26;13:776. doi: 10.1186/1471-2458-13-776 (PMC3765738; doi:10.1186/1471-2458-13-776)

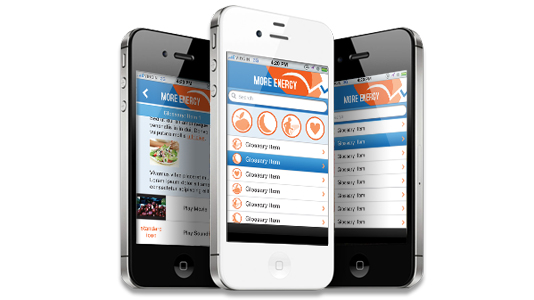


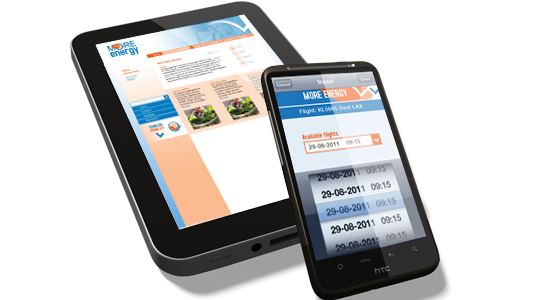


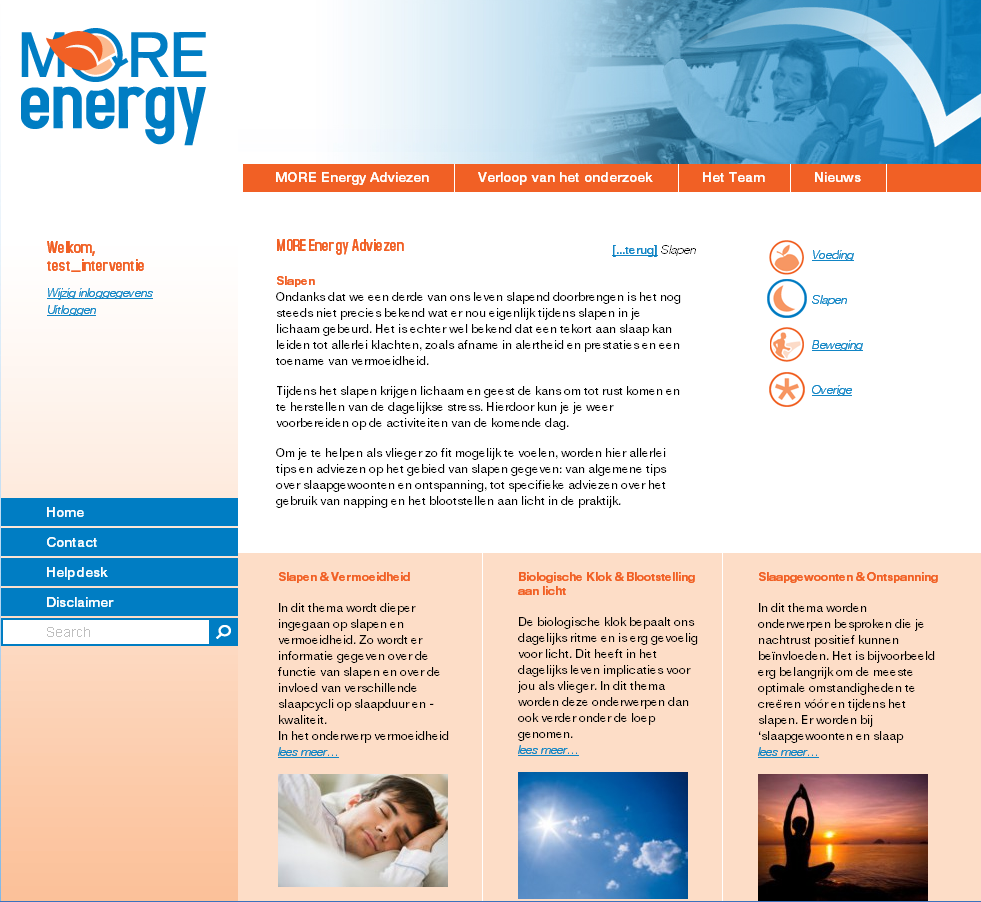

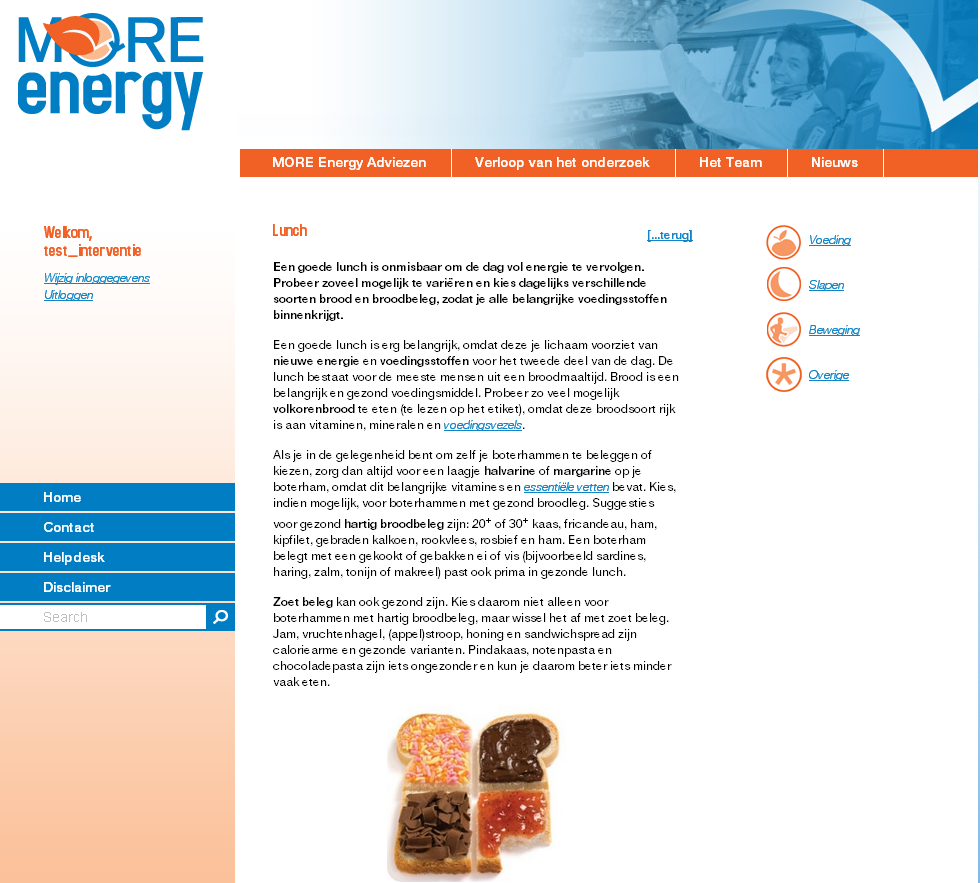

Supplement: Additional file 1 — Examples of the MORE Energy application and website. [file 1471-2458-13-776-S1.doc]
